# Interactions of Nanoparticles with Macrophages and Feasibility of Drug Delivery for Asthma

**DOI:** 10.3390/ijms23031622

**Published:** 2022-01-30

**Authors:** Sung Hun Kang, Yoo Seob Shin, Dong-Hyun Lee, Il Seok Park, Sung Kyun Kim, DongHun Ryu, YongKeun Park, Soo-Hwan Byun, Jeong-Hee Choi, Seok Jin Hong

**Affiliations:** 1 Department of Biomedical Sciences, Hallym University College of Medicine, Chuncheon 24252, Korea; malice23@nate.com; 2 Department of Allergy and Clinical Immunology, Ajou University School of Medicine, Suwon 16499, Korea; drsys93@naver.com (Y.S.S.); ggarisma2@gmail.com (D.-H.L.); 3 Department of Otorhinolaryngology-Head and Neck Surgery, Hallym University College of Medicine, Dongtan Sacred Heart Hospital, 7, Keunjaebong-gil, Hwaseong-si 18450, Korea; ispark@hallym.or.kr (I.S.P.); madein811022@gmail.com (S.K.K.); 4 Department of Physics, Korea Advanced Institute of Science and Technology (KAIST), Daejeon 34141, Korea; donghun.ryu29@gmail.com (D.R.); moofaca@gmail.com (Y.P.); 5 Tomocube, Inc., Daejeon 34051, Korea; 6 Department of Oral & Maxillofacial Surgery, Dentistry, Hallym University College of Medicine, Hallym Sacred Heart Hospital, Anyang 14068, Korea; purheit@daum.net; 7 Department of Pulmonology and Allergy, Hallym University College of Medicine, Dongtan Sacred Heart Hospital, 7, Keunjaebong-gil, Hwaseong-si 18450, Korea; 8 Allergy and Clinical Immunology Research Center, Hallym University College of Medicine, Chuncheon 24252, Korea

**Keywords:** nanoparticles, macrophages, drug delivery, asthma, lipid droplet, 3D holotomography

## Abstract

Understanding the interaction between nanoparticles and immune cells is essential for the evaluation of nanotoxicity and development of nanomedicines. However, to date, there is little data on the membrane microstructure and biochemical changes in nanoparticle-loaded immune cells. In this study, we observed the microstructure of nanoparticle-loaded macrophages and changes in lipid droplets using holotomography analysis. Quantitatively analyzing the refractive index distribution of nanoparticle-loaded macrophages, we identified the interactions between nanoparticles and macrophages. The results showed that, when nanoparticles were phagocytized by macrophages, the number of lipid droplets and cell volume increased. The volume and mass of the lipid droplets slightly increased, owing to the absorption of nanoparticles. Meanwhile, the number of lipid droplets increased more conspicuously than the other factors. Furthermore, alveolar macrophages are involved in the development and progression of asthma. Studies have shown that macrophages play an essential role in the maintenance of asthma-related inflammation and tissue damage, suggesting that macrophage cells may be applied to asthma target delivery strategies. Therefore, we investigated the target delivery efficiency of gold nanoparticle-loaded macrophages at the biodistribution level, using an ovalbumin-induced asthma mouse model. Normal and severe asthma models were selected to determine the difference in the level of inflammation in the lung. Consequently, macrophages had increased mobility in models of severe asthma, compared to those of normal asthma disease. In this regard, the detection of observable differences in nanoparticle-loaded macrophages may be of primary interest, as an essential endpoint analysis for investigating nanomedical applications and immunotheragnostic strategies.

## 1. Introduction

Nanomedicine is the application of nanotechnology for the diagnosis, prevention, and treatment of diseases. Generally, nanoparticles are designed to improve the biodistribution of encapsulated drugs by delivering them more effectively and selectively to the pathological site, also known as site-specific drug delivery. The aim of this strategy is to balance the therapeutic efficacy and toxicity of the compounds. However, the physiological barrier of an interendothelial passage is less than 3 nm; for this reason, many strategies (including nanoparticles) cannot be transferred across impermeable barriers to facilitate treatment at sites of tissue injury [1].

Cell-based delivery systems are a strategy for therapeutic and diagnostic applications that have potential advantages, such as increased bioavailability, improved pharmacokinetics, and reduced toxicity. In particular, blood cells (including erythrocytes, leukocytes, and platelets) are being used as candidates for specific applications, such as circulating lifespan, targeted release ability, and natural adhesion [2]. In general, macrophages can respond rapidly to inflammation through activation, adhesion, and migration via intercellular pathways. Thus, macrophages loaded with therapeutic components can be excellent mediators facilitating the delivery of these agents across the endothelial barrier and releasing drugs to specifically targeted tissues [1].

Understanding the inflammatory and toxicology of artificial nanoparticle interactions with macrophages is the basis for evaluating particle safety, in terms of activation of innate/inflammatory responses and possible uses for nanomedicine [3]. However, to date, there is little data on the effect of nanoparticles on the morphology, especially the membrane microstructure and biomechanical properties, of RAW264.7 macrophages. Information on the interaction between these nanoparticles and macrophages may be important for the evaluation of nanotoxicity and development of nanomedicines. Possible alterations in immune function, following interactions with nanoparticles, could improve our understanding of the defense capabilities of exposed organisms, increasing our knowledge of infections and diseases.

Not only the biochemical changes but also the observable differences could be key factors for systematic understanding the interaction of nanoparticles with macrophages. Lipid droplets are one of the observable cellular organisms that regulate inflammatory responses and phagocytosis. When macrophages ingest and eliminate pathogens through phagocytosis, they require dynamic changes, such as membrane fusion and division. Lipid synthesis provides essential lipids to maintain the cytoskeletal network and plasma membrane, thereby enhancing phagocytosis. Lipid droplets also support phagocytosis by providing phospholipid stores that can be rapidly recruited during membrane expansion [4].

Microscopy analysis has been commonly suggested for lipid droplet characterization; however, fluorescent dyes, which visualize lipid droplets, may cause morphological changes, due to phototoxicity and photobleaching. To overcome this limitation, many studies have attempted to investigate lipid droplets, using various techniques, such as Raman scattering, electron, and atomic force microscopy (AFM)-based approaches [5]. Recently, holotomography, also known as three-dimensional (3D) quantitative phase imaging (QPI), has proposed label-free, high-resolution imaging in living cells [6]. Previously, lipid accumulation in hepatocytes was observed from the reconstructed three-dimensional (3D) refractive index (RI) map of hepatocytes [7,8]. Recently, the cytotoxic effects of positively-charged polystyrene nanoparticles on human alveolar cells were also studied [9].

Asthma is an airway inflammation-related disease, characterized by airway hyperresponsiveness (AHR). Various chemical drugs, peptides, and genetic molecules are used as drug therapies for chronic lung diseases, including asthma. However, most chronic lung diseases cannot be completely cured with medication alone; for this reason, controlling symptoms with steroids, bronchodilators, pirfenidone, or nintedanib is the best option [10]. Conventional drug treatment is usually limited by low drug penetration and response to treatment. To solve this, nanoscale carriers have been used as an advanced strategy to improve the pharmacokinetics of therapeutic agents [10].

Currently, macrophage cell carriers are widely used as in vitro and in vivo inflammation-related models for drug delivery systems owing to their unique properties. In particular, macrophages in cancer target delivery areas known as tumor-associated macrophages (TAMs) show outstanding ability and are frequently found within tumor microenvironments, including hypoxic, necrotic, and inflammatory areas, suggesting that leukocytes can be used to access the microenvironment deep into the tissue [1].

Alveolar macrophages are one of the immune cells involved in the development and progression of asthma. Studies have shown that macrophages are implicated in the maintenance of lung homeostasis and are an essential part of the mechanisms that maintain asthma-related inflammation and tissue damage [11]. Therefore, macrophage-based systems may be applied as an asthma target delivery option. Macrophages also have phagocytic capacity, which can enhance their ability to encapsulate drugs and therapeutic particles.

In this study, we used holotomography analysis to observe the morphological, ultrastructural, and lipid droplet changes. Quantitative measurements of morphological and biophysical parameters in live unlabeled macrophages, compared to monocytes (RAW264.7 cells) provide nanoscale insights into this in vitro model of inflammation. In addition, using RI-based quantitative analysis, observable changes in 3D correlation imaging were used to identify macrophage and nanoparticle interactions. Furthermore, the target delivery efficiency of gold nanoparticle (AuNP)-loaded macrophages was investigated at the intracellular level using ovalbumin (OVA)-induced asthma mouse models. In this regard, the detection of observable differences in nanoparticle-loaded macrophages may be of primary interest as an important endpoint analysis for investigating nanomedical applications and immunotheragnostic strategies.

## 2. Results

We stimulated macrophages using lipopolysaccharide (LPS), which is a major outer membrane component of gram-negative bacteria that plays an important role in the pathogenesis of gram-negative bacterial infections.

**Figure 1:**
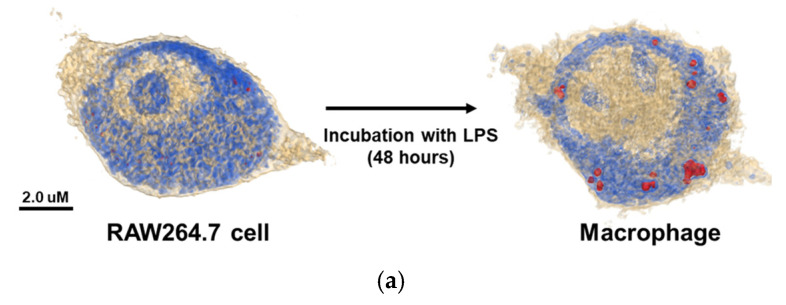

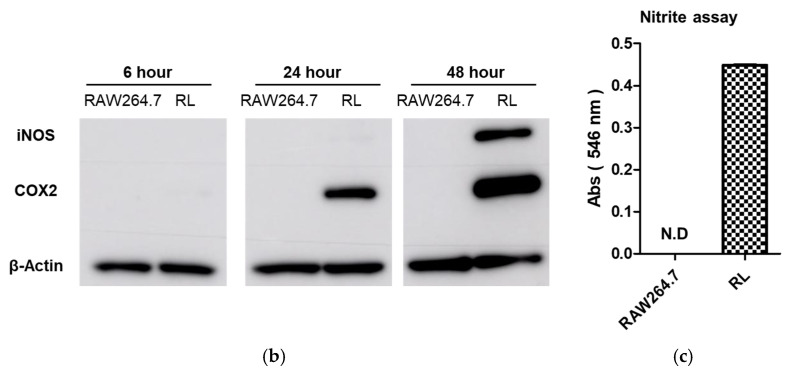
(**a**) The 3D holotomographic images show RAW264.7 cells and LPS-induced macrophages by RI-based imaging technology. When RAW264.7 cells differentiated into macrophages, lipid droplets appeared inside the cells. (Red dots indicate lipid droplets) (**b**) Time-dependent protein expression level of LPS-induced macrophages were analyzed by Western blotting. iNOS and COX2 proteins showed that LPS was activated into RAW264.7 cells to induce macrophages. Β-actin was used as a control. (**c**) Nitrite production in the cell media under LPS treatment was measured by the Griess assay. Nitrite production indicates that iNOS protein induces inflammation in macrophages. (RL: LPS treated RAW264.7 cell).

Inducible nitric oxide synthase (iNOS) and cyclooxygenase-2 (COX-2) proteins are widely recognized as validation tools for LPS-induced macrophage differentiation. Expression of these proteins confirmed macrophage differentiation induced by LPS (Figure 1b). RAW264.7 cells did not express iNOS or COX-2, but COX-2 protein was expressed in cells after 24 h incubation with LPS. During the last 24 h of culture, the expression of COX-2 increased with that of iNOS.

The production of nitric oxide is important for regulating proinflammatory responses in inflammatory diseases. For this reason, we investigated nitric oxide production in LPS-stimulated macrophages. LPS-induced macrophages showed significant nitric oxide production compared to RAW264.7 cells. iNOS protein is responsible for inducing nitric oxide production in macrophages (Figure 1c).

The 3D holotomographic images of LPS-induced macrophages showed significant changes compared to those of untreated RAW264.7 cells (Figure 1a, Video S1, S2), and these morphological changes were confirmed after 48 h of LPS stimulation. RAW264.7 cells exhibited a classical oval shape with a volume of 1071.3 µm^3^. Additionally, the control cell membrane was relatively smooth, with few surrounding lamellipodia. However, LPS-exposed macrophages were larger than control cells, with a cell volume of 1269.5 μm^3^, and the volume of the LPS-induced nuclei was also increased. In particular, the lamellipodia and filopodia of macrophages became much stronger with some dendritic pseudopods. Meanwhile, protein concentration showed negligible changes between LPS-treated and untreated cells. The cell dry mass showed a slight increase, following the change in cell volume.

**Figure 2:**
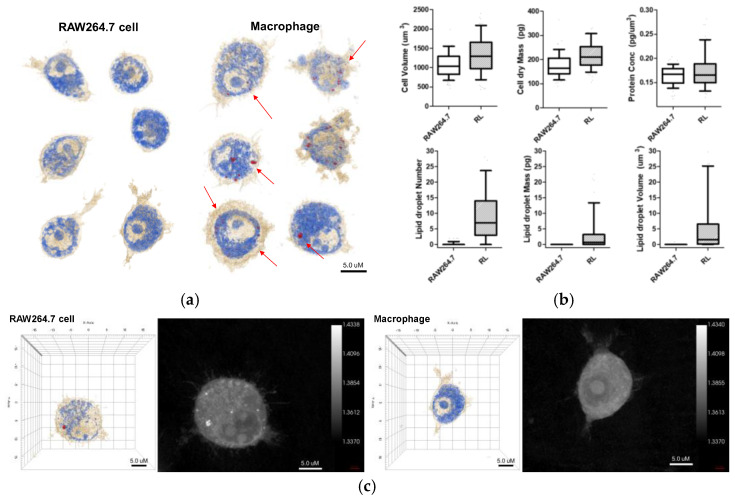
(**a**) 3D holotomographic images of LPS-induced macrophages show significant changes compared to RAW264.7 cell line. Cell size and morphological changes appeared after 48 h of incubation with LPS. (**b**) The characteristics of RAW264.7 cells and macrophages were quantitatively compared through numerical analysis. This analysis results exhibit the morphological and biological changes using RAW264.7 cell (*n* = 320) and macrophage (RL; *n* = 262). Data are presented as a box-and-whiskers plot, with 10–90 percentile of results. Additionally, all of the data show significant differences (*p* < 0.001). (**c**) 3D RI tomograms (left) of each cell were reconstructed from multiple 2D hologram images (right).

Lipid droplet formation is a phenomenon that occurs after LPS treatment and is an important factor. When macrophages were treated with LPS, lipid droplets emerged inside the cells. Because the RI values of lipid droplets are distinctly higher than that of cytoplasm, the lipid droplets are segmented by thresholding a RI value in a measured RI tomogram [7]. The characteristics of the lipid droplets were quantitatively compared using numerical analysis of areas demonstrating an RI value of 1.390. In Figure 2, several parameters, including number, volume, and dry mass, of lipid droplets in cells, with and without LPS stimulation, are shown. The number, volume, and dry mass of lipid droplets in macrophages were 7.000, 1.562 fL, and 0.689 pg per cell, respectively.

**Figure 3:**
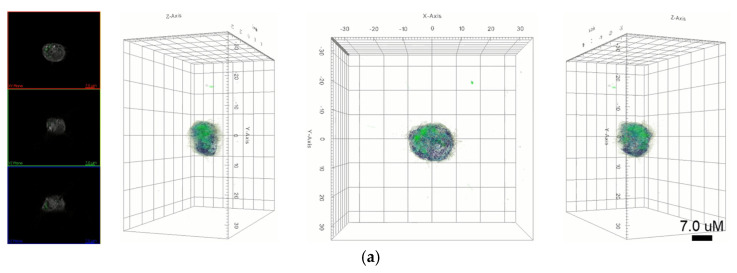

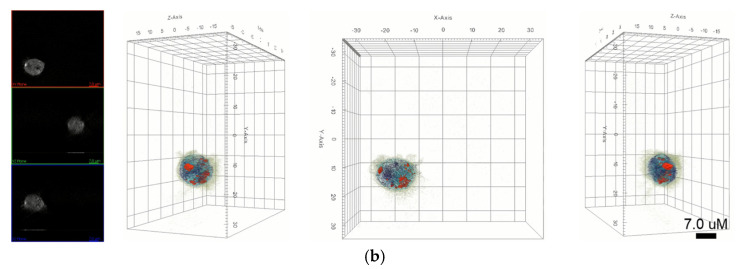
(**a**) 3D holotomography and fluorescence images of GQD-loaded macrophages indicate that GQDs are localized into the cell. Green dots indicate the fluorescence of GQDs. (**b**) 3D holotomographic image of AuNP-loaded macrophage indicate the AuNPs location using an RI-based image system.

In this study, we focused on the interaction of nanoparticles with live macrophages. Two types of nanoparticles were selected, namely AuNPs and graphene quantum dots (GQDs), which represent metal- and carbon-based nanoparticles, respectively. These two nanoparticles show excellent properties, such as biocompatibility and nontoxicity. We performed in vitro biodistribution imaging technology, which can be used to analyze the degree of bonding between nanoparticles and macrophages using 3D holotomographic images. Figure 3 shows the distribution of each nanoparticle in macrophages. AuNPs in holotomographic images were determined by RI detection in the range of 1.3345 to 1.3421 RI values (Figure 3a, Video S3), while GQDs were obtained by fluorescence detection (Figure 3b, Video S4).

**Figure 4:**
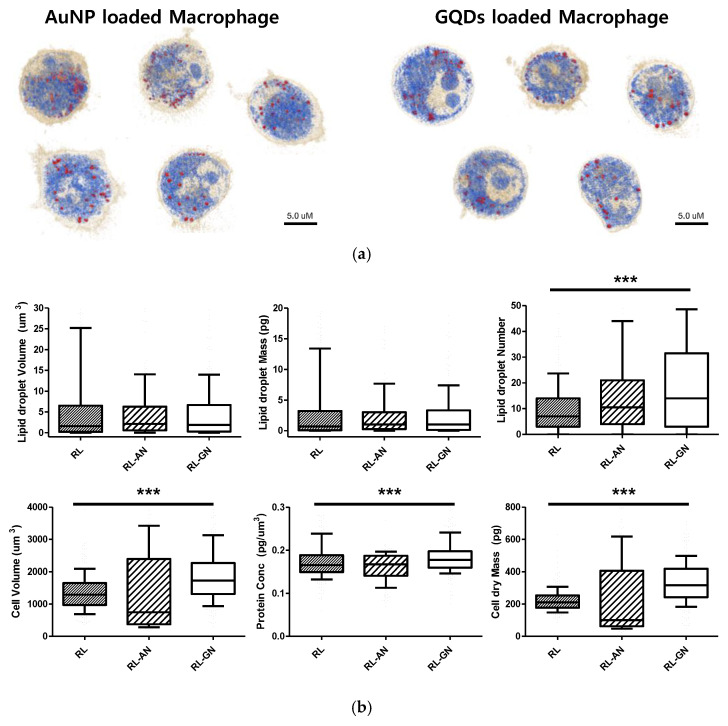
(**a**) 3D holotomographic images show each nanoparticle-loaded macrophage. This image focused on cell cytoplasm and lipid droplet information. (**b**) The properties of nanoparticle-loaded macrophages were quantitatively compared through numerical analysis in lipid droplet number, volume, mass, cell volume, dry mass, and protein concentration. Macrophage (RL; *n* = 262), AuNP-loaded macrophage (RL-AN; *n* = 180), and GQDs-loaded Macrophage (RL-GN; *n* = 233) was used for this analysis. Data are presented as a box-and-whiskers plot, with 10–90 percentile of results. Additionally, the data of lipid droplet number, cell volume, protein concentration, and dry mass showed significant differences (*** represents *p* < 0.0001). For the lipid droplet volume, the mass gave *p*-values of 0.0679 and 0.0672.

Furthermore, as shown in Figure 4, when nanoparticles were absorbed into macrophages, the lipid droplet number and cell volume increased. The volume and mass of the lipid droplets increased slightly, owing to the absorption of nanoparticles. Interestingly, the number of lipid droplets increased more conspicuously than the other factors. The cell volume and dry mass increased because the nanoparticles were loaded into macrophages. Additionally, the filopodium was significantly decreased and showed a smooth and spherical cell shape in both nanoparticle-treated macrophages.

To determine the target delivery capacity of macrophages for asthma-related inflammation, macrophages loaded with AuNPs were applied to healthy and asthmatic mouse models. A gold ion detection method, wherein gold ions indicate the accumulation of macrophages in each organ, was used to track the location of AuNPs. To establish lung-specific inflammatory bodies, we selected an ovalbumin (OVA)-induced asthma model. We used OVA-asthma and OVA-severe models to determine the difference in the level of inflammation in the lungs.

**Figure 5:**
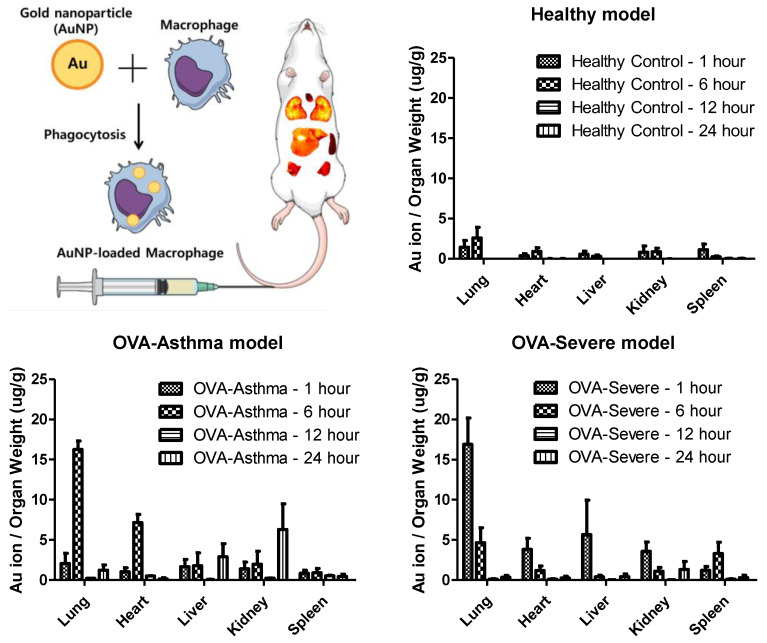
Quantification analysis of gold ion accumulation in the major organs (lung, heart, liver, kidney, spleen), calculated by gold ion per each organ weight. AuNP-loaded macrophage migration exhibited the lung target delivery, and release property.

Figure 5 shows the ex vivo biodistribution in the major organs (lung, heart, liver, kidney, and spleen), based on the gold ion concentration per organ weight. AuNP-loaded macrophages migrated to the lung tissue in the asthma mouse model. Macrophages exhibited a six-fold faster migration into the lung tissue in the OVA-severe model, compared to the OVA-asthma model. These results show that macrophages have increased migratory properties, according to the level of tissue inflammation.

Furthermore, the quantification analysis of gold ion accumulation was performed to analyze the static time level of the AuNP-loaded macrophages in each organ. The maximum accumulation of gold ions was detected in the lungs and released within 24 h.

## 3. Discussion

Cell-based drug delivery systems have been developed to improve pharmacokinetic properties, such as high bioavailability and reduced toxicity of therapeutics. These strategies can achieve advantages, such as prolonged drug release time, nonimmunogenicity, target accumulation, drug release into specialized cellular compartments, and biocompatibility [2]. In particular, macrophages have homing properties to migrate to sites of inflammation and tumors, making them desirable for the development of targeted drug delivery systems. Early studies demonstrated that cells can actively cross the endothelial barrier, including the blood-brain barrier [12]. Therefore, macrophage-mediated delivery systems have been developed to successfully deliver therapeutics to the site of action.

LPS-stimulated RAW264.7 cells have been widely used to prepare macrophage vehicles for drug delivery applications. When LPS stimulation is initiated, the downstream NF-κB signaling pathway is activated to trigger cytokine secretion and nitrite production. During this process, many inflammatory mediators are produced in macrophages, including iNOS, COX-2, and nitric oxide [13]. In particular, nitric oxide is produced by iNOS downregulation, which plays an important role in the innate response of activated macrophages and is involved in immunomodulatory mechanisms [14]. COX-2 is an enzyme that can be induced by inflammatory cytokines and other activators, such as LPS, to release prostaglandin E2 at the inflammation site and is abundant in activated macrophages and other cells in the area. Therefore, COX-2 expression is considered a marker of macrophage activation [15]. Western blot and nitric oxide detection results indicated that LPS stimulation activated the macrophage inflammatory response (Figure 1b,c).

Lipid droplet accumulation is a naturally observed phenotype in infectious, neoplastic, and inflammatory diseases, including cancer. Recent studies have focused on elucidating the function of lipid droplets under physiological and pathological conditions. In particular, the causal relationship regulating the formation of lipid droplets and their functional importance in cell biology is gaining interest [16]. Recent studies have shown that macrophage function is affected by cellular metabolism, including lipid synthesis. Upon stimulation of Toll-like receptor 4, by LPS, macrophages augment lipid synthesis, inflammatory cytokine production, and lipid droplet accumulation. Lipid droplets may support the maintenance of cellular fatty acid homeostasis, production of immunological mediators, and regulation of macrophage functions. As immunological mediators, eicosanoids are key components of immune cell proliferation, activation, and migration [4]. Consequently, lipid droplets are not only involved in lipid storage and transport but may also be involved in inflammation, cell activation, and metabolism, thus becoming markers for macrophage activation.

Previous studies have revealed the morphological and microstructural changes in RAW264.7 cells upon LPS stimulation by AFM analysis. It was found that RAW264.7 macrophages stimulated with LPS increased in size, compared to untreated cells and that morphological changes, such as lamellipodia, were stronger, and the size of the nucleus was increased [13]. Holotomography results are consistent with the results of previous studies, indicating that LPS stimulation induces macrophage activation from RAW264.7 cells (Figure 2). Additionally, holotomography provides more intracellular images than other analytical tools, such as AFM. As shown in Figure 2, lipid droplets were not present in RAW264.7 cells but were significantly revealed by LPS stimulation. Lipid droplets are not robust for detection, owing of their phototoxicity, photobleaching, or crumpling by fixation [17,18]. The advanced technologies in QPI have provided label-free imaging of living cells with quantitative information, including cell volume, cell dry mass, protein concentration, and lipid droplet volume, mass, and number [8]. Based on these results, we confirmed the activation of LPS-stimulated macrophages and differentiated them from RAW264.7, through quantitative information, morphological changes, and the presence of lipid droplets.

Macrophages can phagocytose substances through various mechanisms. Therefore, loading micro- and nanoparticles, such as AuNPs and GQDs, could lead to significant levels of therapeutic efficacy in the field of drug delivery systems [19,20,21]. In addition, drug-loaded macrophages improve the lifespan by evasive renal excretion and/or liver metabolism. Encapsulated drugs are protected from immune responses, including the mononuclear phagocyte system [2,19]. However, the lack of understanding of the interaction between macrophages and engineered nanoparticles hinders the variety of potential for nano-medical applications.

In this study, we identified nanoparticles using two types of systems: AuNPs revealed by RI-based detection and GQDs shown by green fluorescence in macrophages (Figure 3). These were distinguished by observable parameters and quantitative values in macrophages (Figure 4). The results showed significant differences, depending on whether the nanoparticles are loaded, and this may be because phagocytosis requires dynamic changes in plasma membrane fusion and division to take up nanoparticles [22,23]. In particular, lipid droplets are rapidly mobilized for membrane expansion, providing a reservoir of phospholipids to aid phagocytosis [4].

Recent studies have reported that certain nanoparticles induce oxidative stress in cellular conditions, leading to lipid droplet formation. For example, Khatchadourian et al. found that cadmium telluride nanoparticles form lipid droplets and damage intracellular organelles [24]. When the mitochondrial membrane is damaged by oxidative stress or external factors, triglyceride storage increases and lipid droplets accumulate [25,26]. Lipid droplets are motile organelles, and their behavior in stressed cells is dynamic, as lipid metabolism is impaired during oxidative stress. Interestingly, Melo et al. reported that lipid droplets were associated with the phagolysosomes of macrophages during in vivo infection, and some lipid droplets are internalized by phagosomes [27]. Therefore, an increase in lipid droplets may be proposed as a natural response to intracellular stress and phagocytosis. Ultimately, the morphological, structural, and biochemical changes of macrophages by nanoparticle encapsulation are important for understanding and improving drug delivery efficacy. Quantitative values can also help differentiate between them.

Asthma is a chronic disease characterized by persistent inflammation of the airway, and macrophages play an important role in allergen-induced airway inflammation in allergic asthma, such as the phagocytosis of apoptotic cells [28]. Therefore, macrophages can be a strategy for targeted delivery to asthmatic lung tissue, through their inflammatory homing properties. Our study shows the distribution of AuNP-loaded macrophages in a mouse model of asthma with different levels of inflammation. Macrophages show rapid migration and accumulation in the lung tissue, particularly in conditions of chronic inflammatory asthma. The nanoparticle-loaded macrophages leave the site of inflammation after accumulation. This behavior exactly follows the role of macrophages in asthma (Figure 5) [20,28].

As asthma progresses, many inflammatory cells work together with activated resident structural cells to sustain inflammation, through the secretion of proinflammatory mediators, which causes bronchial hyperresponsiveness (BHR), mucus overproduction, and epithelial barrier dysfunction. The resolution of airway inflammation begins with the termination of proinflammatory signaling and granulocyte recruitment. It triggers monocyte recruitment, programmed cell death, or apoptosis through death signaling, which is mediated by natural killer cells. These granulocytes and apoptotic cells are cleared by programmed cell death, with subsequent phagocytosis by the surrounding macrophages [28,29].

This resolution of airway inflammation is activated by the biosynthesis of active endogenous anti-inflammatory and proresolving mediators, which act on key cellular events of inflammation to aid in lung homeostatic progression. In particular, proresolving mediators induce specific alterations in macrophages, such as enhancing phagocytosis of apoptotic cells and shifting from classical to alternative activation of macrophages [29,30]. This recruitment of monocyte and macrophage activity could infer macrophage migration and clearance phenomena in asthmatic lung conditions.

The severe asthma mouse model showed significant augmented responses in airway hypersensitivity, airway inflammation, Th2 cytokine production, and histologic changes, compared to the normal asthma model [31]. In addition, our previous OVA-induced asthma study revealed that increased adhesion molecules, such as ICAM-1 and VCAM-1, were found to influx inflammatory cells from the blood into the lungs [32]. Taken together, the results show that AuNP-loaded macrophages migrate to the target lung tissue in OVA-induced asthma mice, compared to negative control (NC) mice, and demonstrate that these results were significantly facilitated in the severe asthma model. Therefore, our design can be proposed as a targeted delivery strategy for asthmatic lung tissue. Various mechanisms can be applied to the release nanoparticles from macrophages, and photochemical internalization is one of proper modality [33]. Further studies of external stimuli are needed to increase the release of nanoparticles and enhance therapeutic properties of the drug.

## 4. Materials and Methods

### 4.1. AuNP Preparation

AuNPs were prepared following the Turkevich method [34]. The HAuCl4 solution (0.2 mg/mL) was heated at 97.5 °C. with vigorous stirring. When the solution reached a sufficient temperature, trisodium citrate hydrate (4.7 mg) was added and stirred for 30 min. The color of the solution changed from dark blue to red wine with AuNP synthesis. After the reaction, the solution was cooled and stored at 4 °C for subsequent experiments.

### 4.2. GQD Synthesis

The synthesis protocol for GQDs followed that of Md Nafiujjaman [35]. Specifically, 2 g of L-glutamic acid was placed in a glass bottle and heated to 220 °C using a heating mantle. Solid L-glutamic acid turns into a liquid during pyrolysis and then turns brown. Next, 10 mL of distilled water was added to the solution and vigorously stirred for 30 min. The solution was cooled to room temperature and centrifuged at 10,000× *g* for 30 min to collect the supernatant.

### 4.3. Differentiation of RAW264.7 Cells to Macrophages

LPS was used as an inducer of RAW 264.7 cell activation. First, RAW264.7 cells were concentrated to 5 × 10^6^ cells and cultured in a Petri dish containing 2 µg of LPS for 48 h. AuNPs (20 pmol) or GQDs (1 mL of 4 mg/mL) were applied to RAW264.7 cells 24 h after LPS treatment. We used cells incubated with LPS for 48 h, including nanoparticle-treated or untreated samples, for all experiments [20]. Preparation of activated macrophages for holotomographic microscopy analysis followed the RAW264.7 differentiation protocol in TomoDish (Tomodish#1.5 H with a #1.5 H thickness and 50 mm diameter glass bottom; Tomocube, Inc., Daejeon, Korea). All cultures were maintained at 38 °C under 10% CO_2_.

### 4.4. Holotomography

Exploiting holotomography, each sample was subjected to quantitative and qualitative analyses to detect lipid droplets and cell volume, mass, protein concentration, number, and morphology. Holotomography is a laser-based 3D holographic microscopy technique. We used a commercial holotomography system (Tomocube HT-2; Tomocube, Inc.; Daejeon, Korea), which measured both the 3D RI distribution and three-channel 3D fluorescence imaging. The 3D RI tomogram is reconstructed from multiple 2D hologram images, acquired from various illumination angles (49 illuminations and 48 azimuthally symmetric directions). A diode-pumped solid-state laser (*λ* = 532 nm) was used as an illumination source. A beam from the laser is split into two arms using a 2 × 2 fiber. A beam passes through a sample. The diffracted light is collected with a high numerical aperture objective lens (NA = 1.2, UPLSAP 60XW; Olympus, Tokyo, Japan) and then imaged onto a COMOS image sensor (FL3-U3-13Y3MC; FLIR Systems, Wilsonville, OR, USA), which interferes with a reference beam and forms a spatially modulated hologram. A digital micro-mirror device was used to rapidly and precisely control the angle of the illumination [36]. The detailed information on the principle of holotomography, the optical setup, and the reconstuction algorithm can be found elsewhere [37,38,39].

### 4.5. Holotomography

From the reconstructed 3D RI tomograms, the cell body and lipid droplets are segmented by thresholding RI values, using the distinct RI values between surrounding membrane (1.3450–1.3506; beige color), cytoplasm (1.3749–1.3824; blue color), and lipid droplet (>1.3932; red color) [7]. The volume of cell and lipid droplets are directly calculated from the segmentation. The drymass concentration is retrieved using a linear relationship between a RI (*n*) and drymass concentration (c) [40,41], using the priori known RI increment values for cytoplasm (d*n*/d*c* = 0.189) [42] and lipid (d*n*/d*c* = 0.135) [43]. The mass is calculated by multiply the volume and the drymass concentration. We only segmented the volume of lipid droplets based on their distinct RI values and did not segment carbohydrates and nucleic acids for the calculation of cellular drymass [8,44].

### 4.6. Nitric Oxide Assay

Nitric oxide detection in macrophages was performed using a nitric oxide/nitrate colorimetric assay. The supernatant of each sample was mixed with 0.1% Griess reagent (G4410; Merck KGaA, Darmstadt, Germany) and incubated for 5 min. The Griess solution reacts with nitric oxide in the samples to form a purple azo product. The absorbance value of the product was measured at 546 nm with a microplate reader (Epoch; BioTek Instruments, Inc., Winooski, VT, USA) [13].

### 4.7. Western Blot Assay

RAW264.7 cells were treated with LPS, combined with or without nanoparticles (AuNP and GQDs). Total protein was extracted and determined using Bradford reagent (B6916; Merck KGaA), according to the manufacturer’s instructions. Proteins were resolved by 10% sodium dodecyl sulfate-polyacrylamide gel electrophoresis and then transferred onto a nitrocellulose membrane. The membrane was blocked with Tris-buffered saline with Tween-20 (200 mM Tris, 500 mM NaCl, and 0.05% Tween-20) containing 5% skim milk for 1 h and incubated with the primary antibodies. After incubation, the membrane was incubated with a horseradish peroxidase-conjugated secondary antibody (Cell Signaling Technology Inc., Beverly, MA, USA) for 1 h. Antibody-treated proteins were prepared by the reaction with an enhanced chemiluminescent detection solution using a luminescent image analyzer (Amersham Imager 600; GE Healthcare Bio-Sciences AB, Uppsala, Sweden).

### 4.8. Ex Vivo Analysis of the Biodistribution of Nanoparticle-Loaded Macrophages Using BALB/c Asthma Model

Six-week-old female BALB/c mice (Jackson Laboratory, Bar Harbor, ME, USA) were purchased and housed under specific, pathogen-free and OVA conditions. All animal experiments performed in this study were approved by the Institutional Animal Care and Use Committee of Ajou University (IACUC 2019-0020). The mice were divided into three groups, namely NC, OVA-sensitized and challenged (OVA-asthma), and severe OVA-sensitized and challenged groups (OVA-severe). In allergen sensitization and challenge, the mice were first sensitized with intraperitoneal injections of 10 µg of OVA (Thermo Fisher Scientific, Pittsburgh, PA, USA) in 1 mg of alum (Inject Alum; Pierce Biotechnology, Inc., Rockford, IL, USA) on days 0 and 14. Then, sensitized mice were nebulized with 1% or 2% OVA for 30 min in the OVA-asthma and OVA-severe models, respectively, using an ultrasonic nebulizer (NE-Y2; Omron, Kyoto, Japan) on days 28, 29, and 30. Mice in the NC group received only phosphate-buffered saline (PBS). Two days after the last OVA nebulization, the mice were sacrificed by CO_2_ asphyxiation at 1, 6, 12, and 24 h after administration of 1.0 × 10^7^ cells/head of AuNP-loaded macrophages. Organs, such as the heart, lungs, liver, kidneys, and spleen, were harvested, washed with PBS thrice, and homogenized with 0.3% Triton™ X-100 containing PBS. The homogenized sample was mixed with five-fold aqua regia and sonicated for 2 h. Finally, the sample was diluted to 100-fold with double-distilled water and analyzed by inductively coupled plasma mass spectrometry.

### 4.9. Statistical Analysis

Data are presented as a box-and-whiskers plot with 10–90 percentile of results obtained from three independent trials unless otherwise indicated. Analysis of variance (*t*-test) (GraphPad Prism, GraphPad Software, San Diego, CA, USA) was used to determine the statistical significance between RAW264.7, and groups. Analysis of variance was used to determine statistical significance between three or more groups. Statistical significance was set at *p* < 0.05.

## 5. Conclusions

Cell-based drug delivery systems offer several advantages for the development of nanomedicines, and one example is macrophages that deliver therapeutics or engineered particles to the site of inflammation. Changes in observable parameters in nanoparticle-stimulated macrophages are essential for the rapid interpretation of the molecular mechanisms, by which macrophages bind nanoparticles. We investigated the morphological, microstructural, and biochemical changes of macrophages, upon LPS stimulation and nanoparticle encapsulation, which may aid in our understanding of the interactions between nanoparticles and macrophages. We also verified the homing properties of macrophages in an asthma mouse model. Holotomography analysis is a powerful tool for detecting 3D structures and imaging cell morphology at a high resolution that can also provide quantitative data, such as lipid droplet count, volume, mass, and cell characterization. In particular, lipid droplets, revealed in living cells, help provide an understanding of the influence of nanoparticles on macrophages. Therefore, the potential of lipid droplets as markers of disease and targets for novel anti-inflammatory and antineoplastic therapies should be explored. This study supports a novel framework for the visual study of macrophages via holotomography analysis and also demonstrates that holotomography can be used to investigate changes in macrophages upon nanoparticle stimulation at the nanoscale level.

## Data Availability

The data used to support the findings of this study are available from the corresponding author upon request.

## Supplementary Materials

Supplementary Materials are available online at https://www.mdpi.com/article/10.3390/ijms23031622/s1.
